# RNA-seq analysis of the gonadal transcriptome during *Alligator mississippiensis* temperature-dependent sex determination and differentiation

**DOI:** 10.1186/s12864-016-2396-9

**Published:** 2016-01-25

**Authors:** Ryohei Yatsu, Shinichi Miyagawa, Satomi Kohno, Benjamin B. Parrott, Katsushi Yamaguchi, Yukiko Ogino, Hitoshi Miyakawa, Russell H. Lowers, Shuji Shigenobu, Louis J. Guillette, Taisen Iguchi

**Affiliations:** Department of Basic Biology, Faculty of Life Science, SOKENDAI (Graduate University for Advanced Studies), 5-1 Higashiyama, Myodaiji, Okazaki, Aichi 444-8787 Japan; Okazaki Institute for Integrative Bioscience, National Institute for Basic Biology, National Institutes of Natural Sciences, 5-1 Higashiyama, Myodaiji, Okazaki, Aichi 444-8787 Japan; Department of Obstetrics and Gynecology, Medical University of South Carolina, and Marine Biomedicine and Environmental Science Center, Hollings Marine Laboratory, Charleston, SC 29412 USA; National Institute for Basic Biology, National Institutes of Natural Sciences, 38 Nishigonaka, Myodaiji, Okazaki, Aichi 444-8585 Japan; Center for Bioscience Research and Education, Utsunomiya University, 350 Mine-machi, Utsunomiya, Tochigi 321-8505 Japan; Innovative Health Applications, Kennedy Space Center, Merritt Island, FL 32899 USA

**Keywords:** Temperature-dependent sex determination, RNA-seq analysis, *Alligator mississippiensis*

## Abstract

**Background:**

The American alligator (*Alligator mississippiensis*) displays temperature-dependent sex determination (TSD), in which incubation temperature during embryonic development determines the sexual fate of the individual. However, the molecular mechanisms governing this process remain a mystery, including the influence of initial environmental temperature on the comprehensive gonadal gene expression patterns occurring during TSD.

**Results:**

Our characterization of transcriptomes during alligator TSD allowed us to identify novel candidate genes involved in TSD initiation. High-throughput RNA sequencing (RNA-seq) was performed on gonads collected from *A. mississippiensis* embryos incubated at both a male and a female producing temperature (33.5 °C and 30 °C, respectively) in a time series during sexual development. RNA-seq yielded 375.2 million paired-end reads, which were mapped and assembled, and used to characterize differential gene expression. Changes in the transcriptome occurring as a function of both development and sexual differentiation were extensively profiled. Forty-one differentially expressed genes were detected in response to incubation at male producing temperature, and included genes such as Wnt signaling factor *WNT11,* histone demethylase *KDM6B*, and transcription factor *C/EBPA*. Furthermore, comparative analysis of development- and sex-dependent differential gene expression revealed 230 candidate genes involved in alligator sex determination and differentiation, and early details of the suspected male-fate commitment were profiled. We also discovered sexually dimorphic expression of uncharacterized ncRNAs and other novel elements, such as unique expression patterns of *HEMGN* and *ARX*. Twenty-five of the differentially expressed genes identified in our analysis were putative transcriptional regulators, among which were *MYBL2, MYCL,* and *HOXC10,* in addition to conventional sex differentiation genes such as *SOX9*, and *FOXL2.* Inferred gene regulatory network was constructed, and the gene-gene and temperature-gene interactions were predicted.

**Conclusions:**

Gonadal global gene expression kinetics during sex determination has been extensively profiled for the first time in a TSD species. These findings provide insights into the genetic framework underlying TSD, and expand our current understanding of the developmental fate pathways during vertebrate sex determination.

**Electronic supplementary material:**

The online version of this article (doi:10.1186/s12864-016-2396-9) contains supplementary material, which is available to authorized users.

## Background

The intimate interaction between the environment and the organism can be profound; ambient environmental stimuli, such as temperature, are powerful catalysts for biomolecular movement and development that manifest as permanent biological changes. Such is the case for temperature-dependent sex determination (TSD), in which the sexual fates of organisms are seemingly determined not by genotypic factors, but by environmental temperature during a specific embryonic period known as the temperature sensitive period (TSP). In vertebrates, TSD has been observed primarily in reptiles, and the bipotential gonad itself is thought to be receptive to thermal signals [1–3]. However, mechanistic details of this interaction between environmental temperature and TSD transcriptional profile cascade have remained unclear. While many past reports have focused on conserved genes with known function in sexual differentiation across vertebrates, such as *AMH*, *CYP19A1*, and *SOX9* [4–6], very few other genes have been closely investigated, leading to a limited understanding of overall gene expression throughout TSD. Critical questions, such as how the developing gonad translates temperature cues into specific biochemical signals, remain unanswered.

Several hypotheses accounting for the molecular mechanism underlying TSD have been proposed. Steroid hormone involvement has long been suspected to play a role in TSD, as these molecules are known to play a critical role in sex differentiation in non-mammalian vertebrates [7]. Furthermore, glucocorticoid has been demonstrated to induce sex reversal in various vertebrate species including reptiles [8, 9], though how the syntheses of steroid hormones are regulated by temperature remain unanswered. Another major mode of biological response to temperature is epigenetic modification; mounting evidence points toward an involvement of epigenetic modifications in sexual development [10]. Past studies reported sexually dimorphic DNA methylation patterns in promoter regions of major sexual development genes in a temperature specific manner, including TSD organisms [11–13]. Other factors often associated with temperature stress, such as heat shock proteins (*HSPs*) and cold inducible RNA binding proteins (*CIRBPs*), have also been suggested to play a role in TSD [14, 15]. These varying responses to thermal influences at multiple levels ultimately result in a highly receptive regulatory network that underlies multitudes of cellular processes. Thus, the effect of temperature on the molecular environment can be profound and far-reaching, and consequently, there is crucial need to gain a comprehensive picture in order to fully understand the molecular mechanisms underlying TSD.

Crocodilians are thought to be entirely composed of TSD species [16], which includes the American alligator, *Alligator mississippiensis* [17]. In *A. mississippiensis* TSD, the sexual fates of bipotential gonads are directed in a temperature-dependent manner to differentiate into either testes at a male producing temperature (MPT) at 33.5 °C, or into ovaries at female producing temperature (FPT) at 30 °C or 34.5 °C [16, 18]. A recent report shows that sex determination in alligator embryo is thermosensitive as early as stage 15 (Ferguson developmental stage), approximately 18-20 days after oviposition [19, 20]. Prior to this report, however, the TSP was defined as occurring during stages 21-24, roughly between 31 and 46 days after oviposition. It is during this later period (stages 21-24) that temperature effects are reflected in the gonadal development, and many of the key sex determination/differentiation genes acquire sexually dimorphic expression patterns [4–6, 16, 19]. The timing of developmental stages and whole-body morphological growth is also greatly influenced by temperature during embryogenesis in the long term [21]. Unfortunately, an effective method to perform gene manipulation is not currently feasible in alligators, and options are limited for detailed studies. This holds true with many of other reptiles and hence, much of the sex determination mechanisms studies in this evolutionary pivotal clade has been primarily resolved through comparative analyses with mammalian or avian sex determination mechanisms.

With rapidly emerging next generation sequencing technologies, global transcriptome studies such as high-throughput RNA sequencing (RNA-seq) is becoming readily available for non-model organisms [22, 23]. In addition, a number of reptilian genomes have become publicly available [24–26], and comprehensive annotated crocodilian genome assemblies have been released on the National Center for Biotechnology Information (NCBI) [24, 27]. As one of the first TSD species with a published genome, the alligator *A. mississippiensis* is an ideal species for studying molecular signaling cascades and gene expression networks during sex determination in TSD species.

In this study, RNA-seq analyses were performed on developing alligator embryonic gonads incubated under MPT or FPT conditions and sampled at various time points to assess transcriptome changes related to each temperature condition during gonadal differentiation. We present an initial investigation into the sexual development cascade within the alligator TSD system, and provide descriptive data on expression patterns during early sexual development, with the emphasis on the identification of novel candidate genes that might account for alligator sex determination. To our knowledge, this is the first whole transcriptome analysis performed on a TSD organism. These results should allow for insights into the early progression of testis and ovarian fate, and provide a foundation for better understanding the genetic programs driving vertebrate TSD.

## Results and discussion

### Experimental design and sequence assembly

**Fig. 1:**
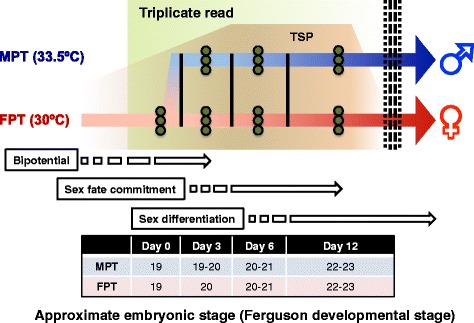
Experimental design. Experimental design of the RNA-seq analysis is illustrated. Bipotential, sex fate commitment and sex differentiation period are indicated, with temperature sensitive period (TSP; indicated in light brown). The dotted line represents the end of the TSP. Eggs were first incubated under female producing temperature (FPT; indicated in red) until just prior to the onset of sexual differentiation (stage 19; Day 0), which were then either shifted to male producing temperature (MPT; indicated in blue) or kept at FPT. Gonadal regions were sampled from individuals at several subsequent time points (Day 3, 6, 12) with corresponding approximate developmental stage (Ferguson) displayed in the bottom table. Day 0–12 represents the timing of sexual differentiation, and three individuals per temperature condition per time points are used

For sample preparation for transcriptome analyses, field collected *A. mississippiensis* eggs were transported to the laboratory and incubated under FPT (30 °C) until Ferguson developmental stage 19 [20], a period in which the gonads are still bipotential and morphologically indistinguishable. At stage 19, a subset of eggs was shifted to MPT (33.5 °C) while the remaining eggs were maintained at FPT for the subsequent incubation period (Fig. 1). Incubations at high FPT (34.5 °C) were not performed in the current study. Tissues comprising the developing gonad were carefully dissected at multiple time points after stage 19; at Day 0, 3, 6, and 12 post-stage 19 for analysis on sex determination cascade. The samples represent the bipotential gonad prior to incubation temperature shift (Day 0), post temperature shift (Day 3), and during sexual fate commitment and differentiation (Day 6, 12). Although the embryos are incubated under different temperatures, the embryonic staging indicate that at least until Day 12 the embryos at respective time points are at within same developmental stage, and confounding variables by differential temperature effects are minimized [28]. In addition to Day 0-12 (sex determination phase), gonads from Day 18, 24, 30, and 36 post-stage 19 were also sampled (sex differentiation phase), although Day 36 was sampled in FPT only (Additional file 1). Illumina HiSeq2500 sequencing produced a total of 375.2 million paired-end reads (2 × 101 bp) and were assessed for quality. The reads were mapped to the alligator genome assembly (allMis0.2), using the latest NCBI annotation available at the time (NCBI *Alligator mississippiensis* Annotation Release 100). The average mapping rates was 88.3 %, among which 94.8 % of the mapped reads were single hits, indicating an overall relatively good quality of reads.

### Transcriptome characterization of alligator gonadal development

**Fig. 2:**
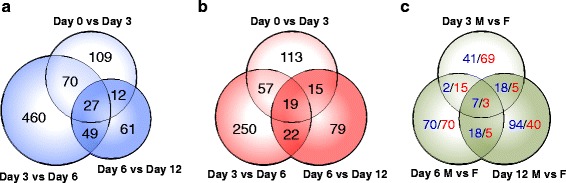
Overlap of development-dependent and sex-dependent differentially expressed genes. Venn diagram of differentially expressed genes (DEGs) in (**a**) Day 0 to Day 12 MPT (indicated in blue) conditions and (**b**) Day 0 to Day 12 FPT (indicated in red) conditions based on genome mappings of Tophat. **c** Venn diagram for number of DEGs between Day 0 to Day 12 MPT and FPT conditions at respective time points, based on genome mapping using Tophat. Number values in blue indicate the number of genes with MPT-biased expression, while values in red indicate the number of genes with FPT-biased expression. All DEGs were determined based on statistical significance (FDR < 0.01) using Cuffdiff software. Further details are available in Additional files 3 and 4

Differentially expressed gene (DEG) analysis was performed using Cuffdiff workflow (ver 2.2.1) [29, 30] to screen DEGs with false discovery rate (FDR) adjusted *p*-value <0.01. To evaluate differential expression across development for embryos incubated at the same temperature, multiple comparisons of the fold differences were conducted between temporally adjacent time points under each incubation condition (MPT/FPT). Development-dependent DEG analysis revealed extensive gene expression kinetics during the course of gonadal morphogenesis, and were profiled (Fig. 2a, b; Additional files 2a and 3). Overall, at MPT, Day 0-12 showed 788 DEGs, of which 158 (20.1 %) displayed expression movement at multiple occasions. At FPT, Day 0-12 showed 555 total DEGs, of which 113 DEGs (20.3 %) also displayed expression movement at multiple occasions. Several noteworthy gene expression patterns were observed; under both MPT and FPT conditions, the transition from Day 3 to 6 accounted for the majority of DEGs, and nearly half of the total DEGs (460 and 250, respectively) were specific to Day 3-6. This timing corresponds to the onset of morphological differentiation in which the enlargement of presumptive Sertoli cells are observed in the medulla at MPT (Stage 21), and might be an indication of the activation of male and female cascade and gonadal fate commitment [31].

Increases in the magnitude of sexually dimorphic gene expression profiles were then examined in detail. Sex-dependent DEG analysis between MPT and FPT samples were conducted at each corresponding time points in respective groups for intergroup comparisons (Fig. 2c, Additional files 2b and 4). Day 0-12 intersex comparisons revealed a total of 457 DEGs. MPT biased gene expression in each time point gradually increased with the progression of sexual development (68, 97, and 137 DEGs at Day 3, 6, and 12, respectively). However, similar to intrasexual development-dependent DEGs, a majority of sexually dimorphic gene expression was time point specific. Ten genes were consistently sexually dimorphic at all 3 time points (*AMH*, *EIF4A2*, *IFRD1*, *JARID2, KDM6B*, *KRT10*, *LOC102561378, LOC102574081*, *UCP2*).

### Identification of immediate temperature-responsive genes

**Fig. 3:**
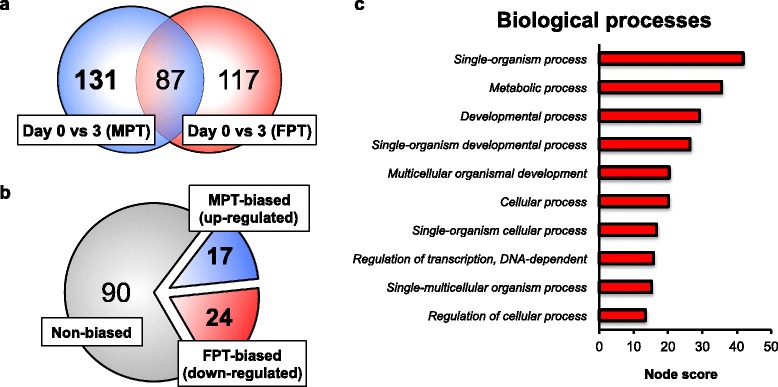
Candidate temperature-responsive differential gene expression. **a** Venn diagrams show the number of DEGs between Day 0 and Day 3 incubated either under MPT (indicated in blue) or FPT (indicated in red). 131 genes (indicated in bold) were found to have MPT-specific gene expression movement, possibly in response to changes in incubation temperature. **b** 41 out of 131 genes were found to have been up- or down-regulated significantly enough to be sexually dimorphic. 17 genes displayed MPT-biased expression and 24 genes displayed FPT-biased expression. Red, blue, and grey indicate FPT-, MPT- or non-bias, respectively. **c** Top 10 biological process gene ontology terms mapped to the candidate temperature-responsive genes with highest node score, based on Blast2GO program

Cross-comparisons between Day 0 FPT embryos and Day 3 at MPT and FPT were performed to evaluate for the MPT-specific immediate temperature-responsive gene expression (Fig. 3). In order to assess the impact of the temperature shift between Day 0 FPT and Day 3 MPT on gene expression, as well as to account for the shared gene expression movement between Day 0-3 FPT and Day 0-3 MPT, significant male-specific differential expressions between Day 0 FPT and Day 3 MPT with resulting sexual dimorphism between Day 3 embryos were identified. The analysis generated 131 MPT specific DEGs (Fig. 3a), of which 41 were also found to be sexually dimorphic between Day 3 MPT and Day 3 FPT as well (Fig. 3b). Seventeen genes with significant upregulation at MPT (*UCP2*, *GALNT5*, *EIF4A2*, etc.) and 24 with significant downregulation (*KDM6B*, *LOC102562106*, *CSRP2*, etc.) were detected, and are likely candidates for immediate temperature-responsive genes (Table 1). While the current study focused upon FPT-to-MPT shift, a complementary MPT-to-FPT study would be ideal to fully identify potential upstream temperature-responsive genes in both male and female sex determination cascades.

**Table 1:** Candidate temperature-responsive genes

Day 0 – Day 3 MPT: MPT dimorphic (up-regulated)				
Description	RefSeq Accession No.	Log_2_(FC)	FDR	Primary ontology
Uncoupling protein 2 (mitochondrial, proton carrier)	XM_006257947.1	1.47	2.63E-03	P: liver regeneration
UDP-N-acetyl-alpha-D-galactosamine:polypeptide N-acetylgalactosaminyltransferase 5	XM_006262802.1	1.46	2.63E-03	C: Golgi membrane
Eukaryotic translation initiation factor 4A2	XM_006262029.1	1.18	2.63E-03	F: translation initiation factor activity
Uroplakin 3A	XM_006265339.1	1.09	2.63E-03	C: integral to membrane
Low-density lipoprotein receptor-related protein 1-like	XM_006265415.1	1.09	2.63E-03	P: lipoprotein transport
Acyl-coenzyme A synthetase ACSM4, mitochondrial-like	XM_006273705.1	1.01	2.63E-03	F: catalytic activity
Solute carrier family 1 (glutamate/neutral amino acid transporter), member 4	XM_006264916.1	0.97	2.63E-03	P: proline transmembrane transport
Transient receptor potential cation channel, subfamily C, member 4 associated protein	XM_006258376.1	0.91	2.63E-03	C: Cul4A-RING ubiquitin ligase complex
CCAAT/enhancer binding protein (C/EBP), alpha	XM_006274301.1	0.90	2.63E-03	P: regulation of cell proliferation
Methylcrotonoyl-CoA carboxylase beta chain, mitochondrial-like	XM_006273834.1	0.86	2.63E-03	F: ligase activity
Myosin XVIIIB	XM_006261765.1	0.86	2.63E-03	C: nucleolus
Chromosome unknown open reading frame, human C1orf63	XM_006276772.1	0.80	2.63E-03	N/A
Interferon-related developmental regulator 1, transcript variant *X*2	XM_006278568.1	0.69	2.63E-03	P: myoblast fate determination
Espin-like	XM_006276123.1	0.65	2.63E-03	N/A
Prenylcysteine oxidase 1	XM_006268735.1	0.62	9.85E-03	C: lysosome
Formimidoyltransferase cyclodeaminase	XM_006260991.1	0.57	6.57E-03	C: cytosol
Uncharacterized LOC102563416	XR_363102.1	infinite	4.73E-03	C: cytosol
Day 0 – Day 3 MPT: FPT dimorphic (down-regulated)				
Description	RefSeq Accession No.	Log_2_(FC)	FDR	Primary Ontology
Lysine (K)-specific demethylase 6B	XM_006271864.1	-1.42	2.63E-03	P: positive regulation of transcription from RNA polymerase II promoter
Protein Jumonji-like	XM_006270621.1	-1.32	2.63E-03	P: negative regulation of transcription from RNA polymerase II promoter
Protein Jumonji-like	XM_006270617.1	-1.32	2.63E-03	P: negative regulation of transcription from RNA polymerase II promoter
Cysteine and glycine-rich protein 2	XM_006269901.1	-1.31	2.63E-03	P: multicellular organismal development
Myeloid protein 1-like	XM_006275897.1	-1.30	2.63E-03	P: granulocyte differentiation
Protease, serine, 35	XM_006258407.1	-1.24	2.63E-03	C: extracellular region
ADAM metallopeptidase with thrombospondin type 1 motif, 15	XM_006262089.1	-1.24	2.63E-03	F: metalloendopeptidase activity
Cellular retinoic acid binding protein 2	XM_006275867.1	-1.18	2.63E-03	F: retinoic acid binding
Angiopoietin-like 1, transcript variant *X*2	XM_006267260.1	-1.18	2.63E-03	C: extracellular space
Transgelin	XM_006278069.1	-1.14	2.63E-03	F: protein binding, bridging
Dimethylaniline monooxygenase [N-oxide-forming] 3-like	XM_006259136.1	-1.06	2.63E-03	C: endoplasmic reticulum membrane
Elastin	XM_006267934.1	-1.05	2.63E-03	N/A
Uncharacterized LOC102575456, transcript variant X1	XR_363216.1	-1.03	2.63E-03	N/A
Hematopoietic prostaglandin D synthase	XM_006265263.1	-1.01	4.73E-03	C: cytoplasm
Hemoglobin subunit epsilon-like	XM_006259088.1	-0.95	2.63E-03	P: oxygen transport
Matrix-remodelling associated 5	XM_006277556.1	-0.94	2.63E-03	C: extracellular region
Epidermal retinol dehydrogenase 2-like	XM_006277024.1	-0.90	8.24E-03	P: retinal metabolic process
v-myb avian myeloblastosis viral oncogene homolog-like 2	XM_006276553.1	-0.82	2.63E-03	P: spindle assembly involved in mitosis
Protein Wnt-11-like, transcript variant *X*2	XM_006265079.1	-0.76	4.73E-03	P: positive regulation of apoptotic process
Thrombospondin 2, transcript variant X1	XM_006277394.1	-0.73	2.63E-03	C: basement membrane
Protein NEL-like	XM_006272242.1	-0.66	2.63E-03	F: kinase activity
Platelet-derived growth factor D-like	XM_006268644.1	-0.63	4.73E-03	P: regulation of peptidyl-tyrosine phosphorylation
RAS-like, family 11, member B	XM_006261252.1	-0.63	6.57E-03	P: small GTPase mediated signal transduction
Ubiquitin carboxyl-terminal esterase L1 (ubiquitin thiolesterase)	XM_006258484.1	-0.58	4.73E-03	F:cysteine-type endopeptidase activity

Oxidative stress responsive-gene uncoupling protein-2 (*UCP2*) displayed the most prominent up-regulation, and suggests the presence of oxidative stress signaling in gonads incubated at MPT [32]. Oxidative stress can be induced by number of factors including the thermosensitive cation channel *TRPV4*, which incidentally is also tightly co-localized with *UCP2* in mammals [33–35]. Interestingly, *TRPV4* has also recently been observed to potentially be involved with alligator TSD [36] and may partially account for the immediate temperature-responsive DEGs identified in the current study. Interestingly, other DEGs identified in this study (e.g. *UPK3A*, *C/EBPA, ESPN*, etc.) are also co-expressed with *TRPV4* in other mammalian tissues, and may share a functional pathway [37–39]. However, the relationship shared between these genes and *TRPV4* during the alligator gonadal sex determination is yet to be elucidated.

Gene ontology (GO) analysis was performed on the temperature-responsive transcripts. Interestingly, ‘Regulation of transcription, DNA-dependent’ was one of the highest biological process terms highlighted, and several transcriptional regulators were detected (Fig. 3c). Most were downregulated by the temperature shift to MPT, such as *KDM6B* and *JARID2*. The roles of chromatin remodelers such as *Cbx2* and *Jmjc1* have been well documented in mammalian sex determination [40, 41], which prompt us to speculate that similar chromatin modification might occur during alligator TSD. Further analysis using Chromatin Immunoprecipitation (ChIP) techniques may help elucidate the chromatin state in alligator gonads during TSD. *WNT11*, which is expressed in mammalian granulosa cells, was also downregulated by shifts to MPT [42]. Transcription factor CCAAT/enhancer binding protein α (*C/EBPA*) was upregulated by shifting incubation temperature to MPT. *C/EBPA* is observed to be active in a wide array of cell differentiation cascades, including mammalian germ cell sex differentiation [43]. Because the current study did not distinguish between cell types, gene expression specific to somatic and primordial germ cell sex development is unclear. Vertebrate somatic and germ cell sex determination cascades are distinct. Thus, resolving the spatial expression patterns for these genes in alligators will further aid in characterizing their roles and functions during TSD.

### Characterization of known sexual development genes

Although vertebrate upstream sex determination mechanisms differ by species, downstream sex differentiation genes appear to be highly conserved by comparison [4], and expression pattern of genes that have been well characterized in alligator sex differentiation were investigated (Additional file 5). Overall, our RNA-seq data was in accordance with the previous reports, based mostly on studies utilizing RT-PCR and *in situ* hybridization techniques. Although, a few surprising discrepancies were revealed by the precise transcript level measurements afforded by RNA-seq analysis [11, 44, 45]. For example, the timing of sexual dimorphism in *AMH* expression was earlier than previously thought, and was observed as early as Day 3. There was a significant upregulation of *AMH* under both MPT and FPT conditions between Day 0 and Day 3; however, the degree of upregulation was far greater at MPT (approximately a 7-fold increase) and continued upregulation was observed at later time points, whereas up-regulation in FPT diminished (Additional file 5). Expression of *AD4BP*/*SF1*, a nuclear receptor involved in gonadogenesis and steroidgenic cell differentiation, was reportedly dynamic in alligator during and after sex determination, though depending on the literature, contradicting expression patterns were also reported [6, 44]. Here, our results indicate that *AD4BP/SF1* is initially fairly stable at both temperatures and only later shows an increase in expression, but does not show significant sexual dimorphism at any time point examined. Expression patterns of *FOXL2*, *FGF9*, *LHX9*, *WNT4*, and *RSPO1* were also characterized, last of which was not sexually dimorphic at any time point during Day 0-12. Species differences in the expression pattern of *RSPO1* during ovarian differentiation have been noted in the past [46, 47], and similar to the pattern observed during *Trachemys scripta* gonad sex differentiation [47], there may be a brief female-biased expression between Day 12 and 18 in the alligator.

### Characterization of candidate sexual development genes

**Fig. 4:**
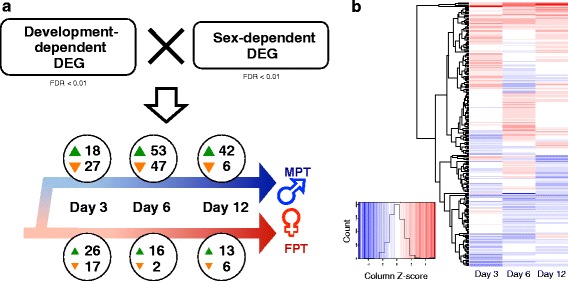
Candidate potential critical genes central for sex determination. **a** Cross comparison between development- and sex-dependent DEGs (FDR < 0.01; Fig. 2) was performed among Day 0 to Day 12, and a total of 230 genes were identified as candidate genes for *A. mississippiensis* sex determination. Green and orange arrows indicate up- and down-regulation, respectively, with number of corresponding genes indicated. The majority of gene expression dynamics associated with development was observed to be MPT-specific. Further information is available in Additional file 6. **b** Hierarchical clustering analysis of sexual dimorphism (M-F log_2_FC) in the candidate genes at Day 3, 6, and 12. Red color indicates high *z*-score (female biased expression) while blue indicates a low *z*-score (male biased expression)

Next, we investigated expression patterns that might provide insight into those genes with central roles in alligator sex determination. Cross-comparative analyses of development-dependent DEGs and sex-dependent DEGs were performed. Differentially expressed genes between sequential time points that also showed sexually dimorphic expression were considered to be potentially critical for gonadal sex determination. This criterion was employed to screen out significant gene expression movement in each MPT and FPT cascades that resulted in sexual dimorphism. With this criterion, 74 female and 172 male upregulated gene candidates (230 genes total) for sexual development were identified (Fig. 4, Additional file 6), including *SOX9*, *AMH*, and *FOXL2*. Genes screened for Day 3 MPT were overall identical to the genes categorized as immediate temperature-responsive genes, with 4 additional genes identified (*AMH*, *FAP*, *COL8A2*, and *COL11A1*). The number of candidate genes increased greatly between Day 3 and Day 6 at MPT, corresponding with the expression of genes involved in testis determination, while only a few candidate genes were identified between Day 3 and Day 6 at FPT.

At MPT, candidate genes identified between Day 3 and Day 6 contained many of the genes with known roles in vertebrate male sexual development, and were profiled for the first time in alligators. These include upregulation of *DMRT3*, *TEX11,* in addition to previously reported *SOX9. PIWIL1* and *TDK*, which are essential for mammalian spermatogenesis [48], were also observed to be up-regulated at MPT during this time. These observations provide details into the transcriptional pathway underlying male fate commitment in alligator, and also reveal genes with potentially crucial roles in somatic and germ cell sex determination/differentiation.

In both the MPT and FPT cascade, several DEG involved with steroid biosynthesis and metabolism were identified, including *HSD17B1*, *STAR*, and *HSD3B2*. Also, some genes expression patterns appeared to have reversed sexually dimorphism between alligator and model organisms. For example, *HEMGN*, a gene critical for male chicken testis development [49], was up-regulated at FPT. *ARX*, a gene involved in mammalian testis development [50] was downregulated on Day 12 at MPT and remained so at later time points. The implications of these gene expression patterns in alligator sex determination are yet to be determined. Finally, 7 uncharacterized transcripts were found to be differentially expressed at various time points. Interestingly, six of these transcripts were identified as ncRNAs (e.g., *LOC102575456*, *LOC102563416*, *LOC102573435*), though their functions and roles during alligator sexual development are yet to be defined. The presence of these ncRNAs may have significant consequences on alligator sex determination as ncRNA (*MHM*) regulation of *DMRT1* plays a pivotal role in *Gallus gallus* sex determination [51]. These observations during the early stages of alligator sexual development highlight both conserved elements and divergences from sex determining mechanisms found in other vertebrates.

### Inference of regulatory network

**Fig. 5:**
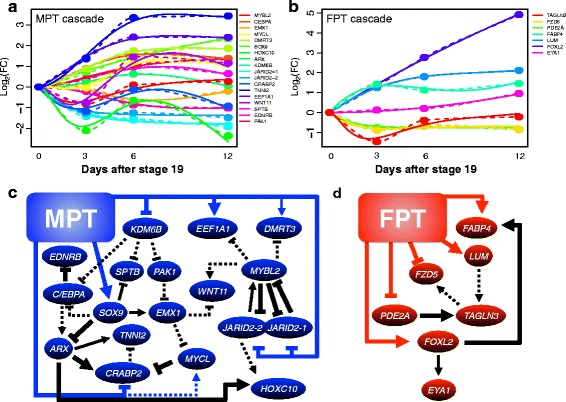
Net*Gene*rator derived model prediction of MPT and FPT cascade. **a**-**b** Interpolated expression (dotted line; dots indicate actual recorded fold change) and inferred patterns derived from the predicted Net*Gene*rator model (solid line) of putative transcriptional regulatory genes over the course of sex differentiation at both (**a**) MPT and (**b**) FPT. **c**-**d** Inferred network model at both (**c**) MPT and (**d**) FPT. Robust (solid line) and non-robust (dotted line) predicted interactions are displayed as gene-gene interactions (black), and temperature-gene interactions (MPT:blue; FPT:red). Thickened lines indicate interaction with high robustness (80 % or above). Following genes names were modified for inference network: LOC102562106 (*JARID2-1*), LOC102561337 (*JARID2-2*), LOC102569158 (*WNT11*), LOC102560544 (*EEF1A1*), LOC102577358 (*DMRT3*), LOC102573123 (*SOX9*), LOC102576325 (*PAK1*), LOC102559361 (*EDNRB*), LOC102563625 (*PDE2A*), and LOC102577040 (*FOXL2*)

Gene ontology analyses of the candidate genes for sexual development recognized 25 genes with putative transcriptional regulatory functions, among which 9 were transcription factors (Table 2, Additional file 7). The gene expression patterns were further characterized as long-term, short-term, or ambiguous depending on the duration of continuous sexual dimorphism during Day 0-12 and Day 18-36. Network modeling of genes from both male and female cascades were performed using the Net*Gene*rator tool [52] (see Materials and Methods) (Fig. 5). The predicted expression patterns based on the modeling overall fit the interpolated expression pattern (Fig. 5a,b), and more than half of the total modeled interactions satisfied the robustness test. The regulatory network model at MPT consisted of 33 edges, 12 robust putative gene-to-gene interactions, and 7 robust putative influences from MPT (Fig. 5c). At FPT, 10 putative edges were constructed with 5 robust putative influences of FPT and 3 robust gene-to-gene interactions (Fig. 5d). As expected, the temperature influences on the regulatory genes were predicted to be widespread in both conditions. In the MPT cascade, *SOX9,* and *ARX* were predicted to be responsible for regulating a number of genes while in the FPT cascade, *FOXL2* was predicted to regulate the phosphatase, *EYA1*, and *FABP4*. Currently, these inferred networks are based on gene expression correlation and generic gene interactions. *In vitro* techniques such as reporter assays will be useful to evaluate such interactions and provide greater insight into the functional relationships between genes in this putative network.

**Table 2:** Candidate critical genes with putative regulatory functions

Candidate transcriptional regulator					
Gene ID	Gene symbol	Description	Molecule type	Expression	Timespan
MPT Day 0 vs MPT Day 3					
XLOC_015643	*CEBPA*	CCAAT/enhancer binding protein (C/EBP), alpha	Transcription factor	Up	Short
XLOC_013275	*KDM6B*	Lysine (K)-specific demethylase 6B	Transcription regulator	Down	Long
XLOC_012144	*LOC102562106*	Protein Jumonji-like	Transcription regulator	Down	Long
XLOC_012145	*LOC102561337*	Protein Jumonji-like	Transcription regulator	Down	Long
XLOC_017186	*CRABP2*	Cellular retinoic acid binding protein 2	Transporter	Down	Short
XLOC_017840	*MYBL2*	v-myb avian myeloblastosis viral oncogene homolog-like 2	Transcription factor	Down	Short
XLOC_006860	*LOC102569158*	Protein Wnt-11-like, transcript variant *X*2	Other	Down	Short
MPT Day 3 vs MPT Day 6					
XLOC_006860	*LOC102569158*	Protein Wnt-11-like, transcript variant *X*2	Other	Up	Short
XLOC_020119	*TNNI2*	Troponin I type 2 (skeletal, fast)	Enzyme	Up	Short
XLOC_009637	*MYCL*	v-myc avian myelocytomatosis viral oncogene lung carcinoma derived homolog	Transcription factor	Up	Short
XLOC_006593	*LOC102560544*	Elongation factor 1-alpha-like	Other	Up	Short
XLOC_003318	*LOC102577358*	Duplex and mab-3 related transcription factor 3-like	Transcription factor	Up	Long
XLOC_013828	*LOC102573123*	Transcription factor SOX-9-like	Transcription factor	Up	Long
XLOC_014546	*LOC102576325*	Serine/threonine-protein kinase PAK 1-like	Protein kinase	Up	Long
XLOC_015643	*CEBPA*	CCAAT/enhancer binding protein (C/EBP), alpha	Transcription factor	Up	Short
XLOC_012336	*SPTB*	Spectrin, beta, erythrocytic, cpytranscript variant X1	Other	Up	Short
XLOC_018228	*LOC102559361*	Endothelin B receptor-like	Receptor	Down	Ambiguous
XLOC_003353	*EMX1*	Empty spiracles homeobox 1	Transcription factor	Down	Short
MPT Day 6 vs MPT Day 12					
XLOC_013828	*LOC102573123*	Transcription factor SOX-9-like	Transcription factor	Up	Long
XLOC_019721	*HOXC10*	Homeobox C10	Transcription factor	Down	Ambiguous
XLOC_001928	*ARX*	Aristaless related homeobox	Transcription factor	Down	Long
FPT Day 0 vs FPT Day 3					
XLOC_003878	*FABP4*	Fatty acid binding protein 4, adipocyte	Transporter	Up	Ambiguous
XLOC_000567	*LUM*	Lumican	Other	Up	Short
XLOC_012101	*TAGLN3*	Transgelin 3	Other	Down	Short
XLOC_007693	*FZD5*	Frizzled family receptor 5	Receptor	Down	Short
XLOC_017000	*LOC102563625*	cGMP-dependent 3′, 5′-cyclic phosphodiesterase-like	Enzyme	Down	Short
FPT Day 6 vs FPT Day 12					
XLOC_001005	*LOC102577040*	Forkhead box protein L2-like	Transcription factor	Up	Long
XLOC_006272	*EYA1*	Eyes absent homolog 1 (Drosophila), transcript variant X1	Phosphatase	Up	Long

## Conclusion

Here, we present the first RNA-seq analysis of gonadal sex determination in a TSD organism. Our analysis clearly shows the dynamic influence of incubation temperature on gene expression, providing insights regarding the state of the gonad at MPT. Differential expression of *UCP2*, *WNT11*, and *KDM6B* highlight the presence of oxidative stress, regulation of Wnt signaling pathway, and chromatin modification on testis development. Furthermore, the global view of gene expression patterns in the gonad during sex determination identified candidate genes that may be integral for the alligator sex determination cascade. Use of network modeling allowed further prediction of the underlying TSD genetic pathway and provides a conceptual framework for empirical tests probing the function of these pathways. These findings, along with the gene expression profiles, will aid future researches on TSD species, and in turn contribute toward further understanding of the vertebrate sex determination mechanisms.

## Methods

### Tissue collection and experimental design

Alligator eggs were collected from five clutches in June of 2010 at Lake Woodruff National Wildlife Refuge, Volusia County, FL, USA under permits from Florida Fish and Wildlife Conservation Commission and the U.S. Fish and Wildlife Service (Permit #: SPGS-10-44). All work involving alligators was performed under the guidelines specified by the Institutional Animal Care and Use Committee at the Medical University of South Carolina (Permit #: 3069). After the eggs were collected from nests, they were transported to the Medical University of South Carolina (Charleston, SC, USA) and incubated under previously established conditions [53, 54]. Rate of embryonic development was predicted based on previous data, and staged according to criteria described by Ferguson [20, 28]. All eggs were incubated under FPT (30 °C) conditions until embryonic stage 19, at which point eggs were split into two incubating temperatures, MPT (33.5 °C) and FPT, and sampled over the course of 36 days. This design was adopted to account for any potential sexual dimorphism present prior to stage 19, and to differentiate between temperature-responsive and developmental genes during the alligator TSD mechanism. The alligator embryos were sampled at various time points starting at stage 19, and 3rd, 6th, 12th, 18th, 24th, and 30th days post-stage 19. Additionally, FPT embryos at 36 days post-stage 19 were also sampled.

### RNA extraction and Illumina library preparation

Gonad-adrenal mesonephros (GAM) complex was first sampled from the alligator embryos and preserved in RNAlater (Ambion/Thermo Fisher Scientific, Waltham, MA, USA) at -20 °C, and later, the gonadal tissue was further dissected from the GAM complex under a dissecting microscope. Total RNA from each individual gonad was extracted using ISOGEN reagent (Nippon Gene, Toyama, Japan) and was purified with Promega SV Total RNA Isolation system (Promega, Madison, WI, USA) according to the manufacturer’s instructions. Qubit RNA assay kit (Life Technologies, Carlsbad, CA, USA), dsDNA HS assay kit (Life Technologies), and Agilent 2100 Bioanalyzer RNA pico kit assay (Agilent Technologies, Santa Clara, CA, USA) was used to assess concentration, DNA contamination, and overall quality. Triplicates were selected from each time point and temperature condition between Day 0 to Day 12 (total of 21 individuals), and single samples were selected from each time point and temperature between Day 18 and Day 36 (total of 7 individuals). Our primary analyses were conducted on the initial phases of sexual determination/differentiation, while single samples taken from Day 18-36 were used to provide an overview for general gene expression kinetics in latter stages. 500 ng of total RNA from each gonad samples was then used for library preparation with Illumina TruSeq RNA sample preparation v2 kit (Illumina, San Diego, CA, USA), following the manufacturer’s instructions. The libraries were then evaluated by using KAPA library quantification kit (Kapa Biosystems, Woburn, MA USA) and 2100 Bioanalyzer High Sensitivity DNA assay (Agilent Technologies). Finally, the multiplexed libraries were pooled into three groups, and sequenced using Illumina HiSeq2500 instrument (Illumina) at National Institute for Basic Biology in Okazaki, Japan. Sequencing was performed as 101 bp, paired-end reads in three lanes. The RNA-seq reads are available through DRA under the accession number: DRA004128-41.

### Differential gene expression analysis

Initial FASTQ files were subjected to quality assessment using FastQC tool (http://www.bioinformatics.bbsrc.ac.uk/projects/fastqc/). Raw reads were then mapped and assembled using the Tuxedo pipeline (Tophat software: ver. 2.0.12; Cufflinks software: ver.2.2.1) [29, 30, 55]. Individual paired–end sequence reads from each sample were aligned to publicly available alligator genome assembly (NCBI database; Assembly name: allMis0.2; Assembly accession: GCF_000281125.1) with supplement gene model annotation (NCBI *Alligator mississippiensis* Annotation Release 100), and directed to report best possible alignment found with ‘-read-realign-edit-dist’ option. The mapping rates for each experimental group were assessed using Samtools ‘flagstat’ command [56]. Differential expression between each condition was tested following the Cufflinks workflow (ver. 2.2.1), and Cuffdiff software. The significance of initial differential expression was tested at significance level α = 0.05, adjusted with allowed FDR at 0.05 following Benjamini-Hochberg correction. DEGs were further screened by FDR at 0.01.

### Gene ontology mapping

Contigs were blasted against the NCBI nr database using blastx program, with a minimum E-value score set to 1.0E-06. Successful blast hit results were then imported to Blast2GO program (ver. 3.1.3) where they were functionally mapped and annotated [57]. Cut-off threshold was set to 55 and the GO level weighting set to 5. Additionally, InterPro IDs from InterProScan were merged to the annotation for further accuracy. ‘Make combined graph’ analysis was performed to score and evaluate the GO distribution in defined gene sets, and top 10 GO terms with highest node scores were selected.

### Statistical analysis and network inference

Basic statistical analysis and graph constructions were conducted using Microsoft Excel, R, and GraphPad Prism software (Version 5.0b; GraphPad Software, Inc., San Diego, CA, USA). Log_2_ transformed FPKM + 1 values were used for both the MA plot and hierarchical clustering heatmap. The Net*Gene*rator tool (ver. 2.4) [52] was used to perform network inference analysis with time-series log_2_ fold change from select candidate DEGs (Table 2) under each incubation temperature condition. Net*Gene*rator offers modeling gene regulatory network from time series data, based on linear differential expression intensities with consideration of influences from external stepwise input signals. Network analysis and robustness analysis was conducted using modified protocol from Schulze et al [58]. In current analysis, input signal was defined as the environmental temperature (MPT or FPT). Prior-knowledge gene-to-gene coexpression data was based on *Mus musculus* (confidence score = 0.25) as obtained from Genemania (genemania.org) and was implemented in the analysis [59–67]. Several parameters were tested and ‘allowedError’ was adjusted at 0.01. Robustness of the network was tested using two methods. In the first test, the inference analysis was repeated 1000 times with artificial Gaussian noise (mean = 0; standard deviation = 0.05) distributed to the expression data to test for susceptibility to perturbations. In second test, the inference analysis was repeated 1000 times with 10 % of the total prior knowledge randomly omitted to test for dependency of the interactions on prior knowledge. Interactions that were predicted more than 50 % of the time in both tests were considered robust.

### Availability of supporting data

The data sets supporting the results of this article are included within the article and its additional files.

## Additional files

Additional file 1:
**Additional experimental design. Experimental design of the additional RNA-seq analysis during sex differentiation period is illustrated.** The dotted line represents the close of the TSP. The eggs were first incubated at female producing temperature (FPT; indicated in red) until just prior to sexual differentiation (stage 19; Day 0), at which point groups of eggs were either shifted to male producing temperature (MPT; indicated in blue) or kept at FPT. Gonadal regions were sampled from individuals at several subsequent time points (Day 18, 24, 30, 36) with corresponding approximate developmental stage (Ferguson) displayed in the bottom table. Day 18-36 represents sex differentiation group, and one individual per temperature condition per time points are used. (PDF 71 kb) Additional file 2:
**Development-dependent DEGs and Sex-dependent DEGs in alligator transcriptomic profiles.** (a) MA plot, with gene expression values expressed as log_2_FPKM and fold change expressed as Log_2_FC, is constructed using differential expression analysis between each time point (Day 0-3, Day 3–6, Day 6-12). (b) MA plot, with gene expression values expressed as log_2_FPKM and fold change expressed as Log_2_FC, is constructed using differential expression analysis results between FPT and MPT (30 °C vs 33.5 °C) for Day 3 FPT vs Day 3 MPT, Day 6 FPT vs Day 6 MPT, and Day 12 FPT vs Day 12 MPT. For both MA plot, 20,181 genes, based on NCBI genome annotation, were examined for differential expression. Red dots indicate significantly up- or down-regulated genes at FDR < 0.01. (PDF 1359 kb) Additional file 3:
**Annotation of development-dependent dimorphic genes in gonad during Day 0–12.** Annotation of development-dependent significantly up- and down- regulated DEGs at FDR < 0.01 in gonadal regions incubated under MPT and FPT conditions during Day 0 to Day 12. Ordered by decreasing fold change. (XLSX 196 kb) Additional file 4:
**Annotation of sexually dimorphic genes in gonad during Day 0–12.** Annotation of significant sexually dimorphic DEGs at FDR < 0.01 in gonadal region between MPT and FPT during Day 0 to Day 12. Ordered by decreasing fold change. (XLSX 98 kb) Additional file 5:
**Expression profiles of major genes involved in sex differentiation.** Expression profiles of various major genes involved in gonadal sex determination in model organisms are displayed based on FPKM values from RNA-seq analysis under FPT conditions (indicated in red) and MPT conditions (indicated in blue). Predicted onset of sexual differentiation is indicated in orange background; * FDR ≤0.01. (PPT 968 kb) Additional file 6:
**Annotation of critical genes involved in sex determination.** Annotation of candidate critical DEGs involved in sex determination during Day 0–12. Ordered by decreasing fold change. DEGs with both significant development-dependent differential expression (Additional file 3) and sex-dependent differential expression (Additional file 4) were screened, as potential critical genes for alligator male and female cascade. (XLSX 64 kb) Additional file 7:
**Expression profiles of candidate transcription factors involved in sex differentiation.** Expression profiles of candidate transcription factors involved in gonadal sex determination are displayed based on FPKM values from RNA-seq analysis under FPT conditions (indicated in red) and MPT conditions (indicated in blue). Predicted onset of sexual differentiation is indicated in orange background; * FDR ≤0.01. (PPT 344 kb)

## Abbreviations

BLAST: basic local alignment search tool
DEG: differentially expressed gene
FC: fold change
FDR: false discovery rate
FPKM: fragments per kilobase of transcript per million fragments mapped
FPT: female-producing temperature
GO: gene ontology
MPT: male-producing temperature
NCBI: National Center for Biotechnology Information
TSD: temperature-dependent sex determination
TSP: temperature-sensitive period

## References

[1] Bull JJ (1980). Sex determination in reptiles. Q Rev Biol..

[2] Bull JJ (1985). Sex determining mechanisms: an evolutionary perspective. Experientia..

[3] Shoemaker-Daly CM, Jackson K, Yatsu R, Matsumoto Y, Crews D (2010). Genetic network underlying temperature-dependent sex determination is endogenously regulated by temperature in isolated cultured *Trachemys scripta* gonads. Dev Dyn..

[4] Morrish BC, Sinclair AH (2002). Vertebrate sex determination: many means to an end. Reproduction..

[5] Kohno S, Parrott BB, Yatsu R, Miyagawa S, Moore BC, Iguchi T (2014). Gonadal differentiation in reptiles exhibiting environmental sex determination. Sex Dev..

[6] Valenzuela N, Neuwald JL, Literman R (2013). Transcriptional evolution underlying vertebrate sexual development. Dev Dyn..

[7] Lance VA (2009). Is regulation of aromatase expression in reptiles the key to understanding temperature-dependent sex determination?. J Exp Zool A Ecol Genet Physiol..

[8] Nakamura M (2010). The mechanism of sex determination in vertebrates-are sex steroids the key-factor?. J Exp Zool A Ecol Genet Physiol..

[9] Warner DA, Radder RS, Shine R (2009). Corticosterone exposure during embryonic development affects offspring growth and sex ratios in opposing directions in two lizard species with environmental sex determination. Physiol Biochem Zool..

[10] Piferrer F (2013). Epigenetics of sex determination and gonadogenesis. Dev Dyn..

[11] Parrott BB, Kohno S, Cloy-McCoy JA, Guillette LJ (2014). Differential incubation temperatures result in dimorphic DNA methylation patterning of the SOX9 and aromatase promoters in gonads of alligator (*Alligator mississippiensis*) embryos. Biol Reprod..

[12] Matsumoto Y, Buemio A, Chu R, Vafaee M, Crews D (2013). Epigenetic control of gonadal aromatase (cyp19a1) in temperature-dependent sex determination of red-eared slider turtles. PLoS One..

[13] Navarro-Martin L, Vinas J, Ribas L, Diaz N, Gutierrez A, Di Croce L (2011). DNA methylation of the gonadal aromatase (cyp19a) promoter is involved in temperature-dependent sex ratio shifts in the European sea bass. PLoS Genet..

[14] Kohno S, Katsu Y, Urushitani H, Ohta Y, Iguchi T, Guillette LJ (2010). Potential contributions of heat shock proteins to temperature-dependent sex determination in the American alligator. Sex Dev..

[15] Rhen T, Schroeder A (2010). Molecular mechanisms of sex determination in reptiles. Sex Dev..

[16] Lang JW, Andrews HV (1994). Temperature-dependent sex determination in crocodilians. J Exp Zool..

[17] Ferguson MW, Joanen T (1982). Temperature of egg incubation determines sex in *Alligator mississippiensis*. Nature..

[18] Ferguson MWJ, Joanen T (1983). Temperature-dependent sex determination in *Alligator mississippiensis*. J Zool..

[19] McCoy JA, Parrott BB, Rainwater TR, Wilkinson PM, Guillette LJ (2015). Incubation history prior to the canonical thermosensitive period determines sex in the American alligator. Reproduction..

[20] Ferguson MWJ, Gans C, Billet F, Maderson PFA (1985). The reproductive biology and embryology of crocodilians. Biology of the Reptilia. 14. Development A.

[21] Deeming DC, Ferguson MWJ (1989). Effects of incubation temperature on growth and development of embryos of *Alligator mississippiensis*. J Comp Physiol B..

[22] Smith S, Bernatchez L, Beheregaray LB (2013). RNA-seq analysis reveals extensive transcriptional plasticity to temperature stress in a freshwater fish species. BMC Genomics..

[23] Sun F, Liu S, Gao X, Jiang Y, Perera D, Wang X (2013). Male-biased genes in catfish as revealed by RNA-seq analysis of the testis transcriptome. PLoS One..

[24] Wan QH, Pan SK, Hu L, Zhu Y, Xu PW, Xia JQ (2013). Genome analysis and signature discovery for diving and sensory properties of the endangered Chinese alligator. Cell Res..

[25] Shaffer HB, Minx P, Warren DE, Shedlock AM, Thomson RC, Valenzuela N (2013). The western painted turtle genome, a model for the evolution of extreme physiological adaptations in a slowly evolving lineage. Genome Biol..

[26] St John JA, Braun EL, Isberg SR, Miles LG, Chong AY, Gongora J (2012). Sequencing three crocodilian genomes to illuminate the evolution of archosaurs and amniotes. Genome Biol..

[27] Green RE, Braun EL, Armstrong J, Earl D, Nguyen N, Hickey G (2014). Three crocodilian genomes reveal ancestral patterns of evolution among archosaurs. Science..

[28] Kohno S, Guillette LJ, Matthiessen P (2013). Endocrine disruption and reptiles: Using the unique attributes of temperature-dependent sex determination to assess impacts. Endocrine disrupters: Hazard Testing and Assessment Methods.

[29] Trapnell C, Williams BA, Pertea G, Mortazavi A, Kwan G, van Baren MJ (2010). Transcript assembly and quantification by RNA-seq reveals unannotated transcripts and isoform switching during cell differentiation. Nat Biotechnol..

[30] Trapnell C, Roberts A, Goff L, Pertea G, Kim D, Kelley DR (2012). Differential gene and transcript expression analysis of RNA-seq experiments with TopHat and Cufflinks. Nat Protoc..

[31] Smith CA, Joss JMP (1993). Gonadal sex differentiation in *Alligator mississippiensis*, a species with temperature-dependent sex determination. Cell Tissue Res..

[32] Chan SH, Wu CA, Wu KL, Ho YH, Chang AY, Chan JY (2009). Transcriptional upregulation of mitochondrial uncoupling protein 2 protects against oxidative stress-associated neurogenic hypertension. Circ Res..

[33] Bubolz AH, Mendoza SA, Zheng X, Zinkevich NS, Li R, Gutterman DD (2012). Activation of endothelial TRPV4 channels mediates flow-induced dilation in human coronary arterioles: role of Ca^2+^ entry and mitochondrial ROS signaling. Am J Physiol Heart Circ Physiol..

[34] Güler AD, Lee H, Iida T, Shimizu I, Tominaga M, Caterina M (2002). Heat-evoked activation of the ion channel, TRPV4. J Neurosci..

[35] Cohen RD, Brown CL, Nickols C, Levey P, Boucher BJ, Greenwald SE (2011). Inbuilt mechanisms for overcoming functional problems inherent in hepatic microlobular structure. Comput Math Methods Med..

[36] Yatsu R, Miyagawa S, Kohno S, Saito S, Lowers RH, Ogino Y (2015). TRPV4 associates environmental temperature and sex determination in the American alligator. Scientific Reports..

[37] Homma Y, Nomiya A, Tagaya M, Oyama T, Takagaki K, Nishimatsu H (2013). Increased mRNA expression of genes involved in pronociceptive inflammatory reactions in bladder tissue of interstitial cystitis. J Urol..

[38] Ovrevik J, Refsnes M, Lag M, Holme JA, Schwarze PE (2015). Activation of proinflammatory responses in cells of the airway mucosa by particulate matter: Oxidant- and non-oxidant-mediated triggering mechanisms. Biomolecules..

[39] Vollrath MA, Kwan KY, Corey DP (2007). The micromachinery of mechanotransduction in hair cells. Annu Rev Neurosci..

[40] Katoh-Fukui Y, Tsuchiya R, Shiroishi T, Nakahara Y, Hashimoto N, Noguchi K (1998). Male-to-female sex reversal in M33 mutant mice. Nature..

[41] Kuroki S, Matoba S, Akiyoshi M, Matsumura Y, Miyachi H, Mise N (2013). Epigenetic regulation of mouse sex determination by the histone demethylase Jmjd1a. Science..

[42] Harwood BN, Cross SK, Radford EE, Haac BE, De Vries WN (2008). Members of the WNT signaling pathways are widely expressed in mouse ovaries, oocytes, and cleavage stage embryos. Dev Dyn..

[43] Ewen K, Jackson A, Wilhelm D, Koopman P (2010). A male-specific role for p38 mitogen-activated protein kinase in germ cell sex differentiation in mice. Biol Reprod..

[44] Western PS, Harry JL, Marshall Graves JA, Sinclair AH (2000). Temperature-dependent sex determination in the American alligator: expression of SF1, WT1 and DAX1 during gonadogenesis. Gene..

[45] Western PS, Harry JL, Graves JA, Sinclair AH (1999). Temperature-dependent sex determination in the American alligator: AMH precedes SOX9 expression. Dev Dyn..

[46] Smith CA, Shoemaker CM, Roeszler KN, Queen J, Crews D, Sinclair AH (2008). Cloning and expression of R-Spondin1 in different vertebrates suggests a conserved role in ovarian development. BMC Dev Biol..

[47] Matsumoto Y, Hannigan B, Crews D (2014). Embryonic PCB exposure alters phenotypic, genetic, and epigenetic profiles in turtle sex determination, a biomarker of environmental contamination. Endocrinology..

[48] Saxe JP, Chen M, Zhao H, Lin H (2013). Tdrkh is essential for spermatogenesis and participates in primary piRNA biogenesis in the germline. EMBO J..

[49] Nakata T, Ishiguro M, Aduma N, Izumi H, Kuroiwa A (2013). Chicken hemogen homolog is involved in the chicken-specific sex-determining mechanism. Proc Natl Acad Sci USA.

[50] Kitamura K, Yanazawa M, Sugiyama N, Miura H, Iizuka-Kogo A, Kusaka M (2002). Mutation of ARX causes abnormal development of forebrain and testes in mice and X-linked lissencephaly with abnormal genitalia in humans. Nat Genet..

[51] Teranishi M, Shimada Y, Hori T, Nakabayashi O, Kikuchi T, Macleod T (2001). Transcripts of the MHM region on the chicken Z chromosome accumulate as non-coding RNA in the nucleus of female cells adjacent to the DMRT1 locus. Chromosome Res..

[52] Weber M, Henkel SG, Vlaic S, Guthke R, van Zoelen EJ, Driesch D (2013). Inference of dynamical gene-regulatory networks based on time-resolved multi-stimuli multi-experiment data applying NetGenerator V2.0. BMC Syst Biol..

[53] Milnes MR, Bermudez DS, Bryan TA, Gunderson MP, Guillette LJ (2005). Altered neonatal development and endocrine function in *Alligator mississippiensis* associated with a contaminated environment. Biol Reprod..

[54] Urushitani H, Katsu Y, Miyagawa S, Kohno S, Ohta Y, Guillette LJ (2011). Molecular cloning of anti-Müllerian hormone from the American alligator, Alligator mississippiensis. Mol Cell Endocrinol..

[55] Trapnell C, Pachter L, Salzberg SL (2009). TopHat: discovering splice junctions with RNA-Seq. Bioinformatics..

[56] Li H, Handsaker B, Wysoker A, Fennell T, Ruan J, Homer N (2009). The sequence alignment/map format and SAMtools. Bioinformatics..

[57] Conesa A, Gotz S, Garcia-Gomez JM, Terol J, Talon M, Robles M (2005). Blast2GO: a universal tool for annotation, visualization and analysis in functional genomics research. Bioinformatics..

[58] Schulze S, Henkel SG, Driesch D, Guthke R, Linde J (2015). Computational prediction of molecular pathogen-host interactions based on dual transcriptome data. Front Microbiol..

[59] Gallardo TD, John GB, Shirley L, Contreras CM, Akbay EA, Haynie JM (2007). Genomewide discovery and classification of candidate ovarian fertility genes in the mouse. Genetics..

[60] Moggs JG, Tinwell H, Spurway T, Chang HS, Pate I, Lim FL (2004). Phenotypic anchoring of gene expression changes during estrogen-induced uterine growth. Environ Health Perspect..

[61] Schreiner CM, Bell SM, Scott WJ (2009). Microarray analysis of murine limb bud ectoderm and mesoderm after exposure to cadmium or acetazolamide. Birth Defects Res A Clin Mol Teratol..

[62] Lattin JE, Schroder K, Su AI, Walker JR, Zhang J, Wiltshire T (2008). Expression analysis of G protein-coupled receptors in mouse macrophages. Immunome Res..

[63] Thorrez L, Van Deun K, Tranchevent LC, Van Lommel L, Engelen K, Marchal K (2008). Using ribosomal protein genes as reference: a tale of caution. PLoS One..

[64] Hernandez-Novoa B, Bishop L, Logun C, Munson PJ, Elnekave E, Rangel ZG (2008). Immune responses to *Pneumocystis murina* are robust in healthy mice but largely absent in CD40 ligand-deficient mice. J Leukoc Biol..

[65] Jacobs JP, Ortiz-Lopez A, Campbell JJ, Gerard CJ, Mathis D, Benoist C (2010). Deficiency of CXCR2, but not other chemokine receptors, attenuates a murine model of autoantibody-mediated arthritis. Arthritis Rheum..

[66] Zapala MA, Hovatta I, Ellison JA, Wodicka L, Del Rio JA, Tennant R (2005). Adult mouse brain gene expression patterns bear an embryologic imprint. Proc Natl Acad Sci USA.

[67] Akerblad P, Månsson R, Lagergren A, Westerlund S, Basta B, Lind U (2005). Gene expression analysis suggests that EBF-1 and PPARγ2 induce adipogenesis of NIH-3 T3 cells with similar efficiency and kinetics. Physiol Genomics..

