# Supplementary figures and images for: RNA-seq analysis of the gonadal transcriptome during Alligator mississippiensis temperature-dependent sex determination and differentiation
Source: BMC Genomics. 2016 Jan 25;17:77. doi: 10.1186/s12864-016-2396-9 (PMC4727388; doi:10.1186/s12864-016-2396-9)

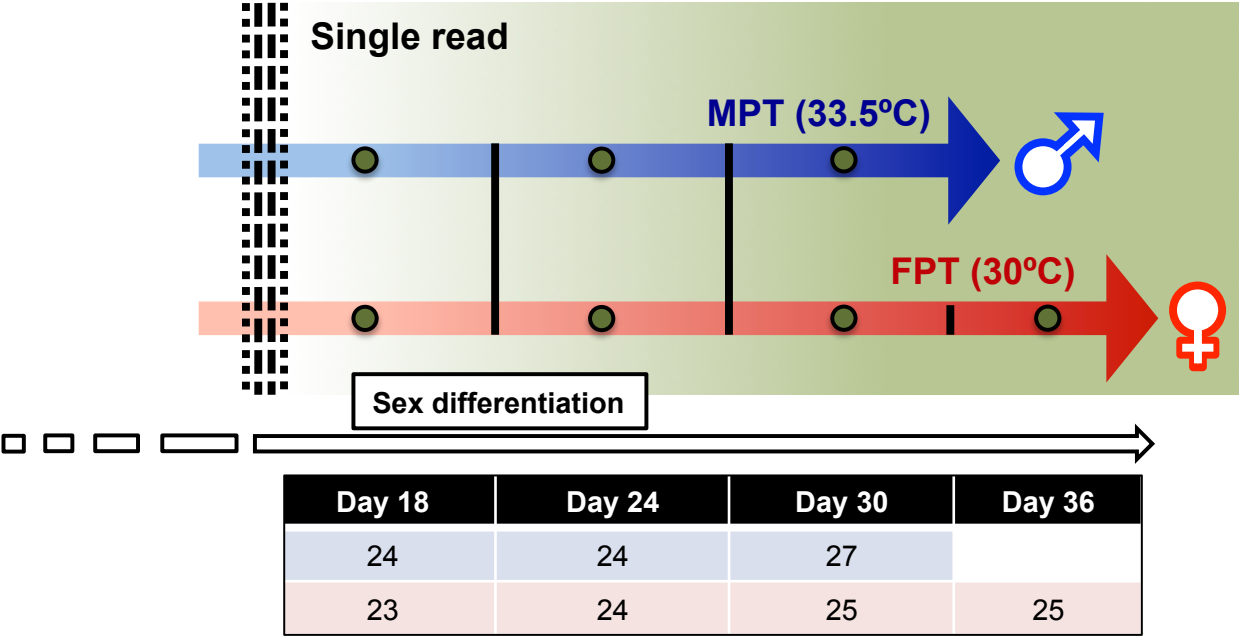

Approximate embryonic stage (Ferguson developmental stage)

Supplement: Additional file 1: — Additional experimental design. Experimental design of the additional RNA-seq analysis during sex differentiation period is illustrated. The dotted line represents the close of the TSP. The eggs were first incubated at female producing temperature (FPT; indicated in red) until just prior to sexual differentiation (stage 19; Day 0), at which point groups of eggs were either shifted to male producing temperature (MPT; indicated in blue) or kept at FPT. Gonadal regions were sampled from individuals at several subsequent time points (Day 18, 24, 30, 36) with corresponding approximate developmental stage (Ferguson) displayed in the bottom table. Day 18-36 represents sex differentiation group, and one individual per temperature condition per time points are used. (PDF 71 kb) [file 12864_2016_2396_MOESM1_ESM.pdf]

**a**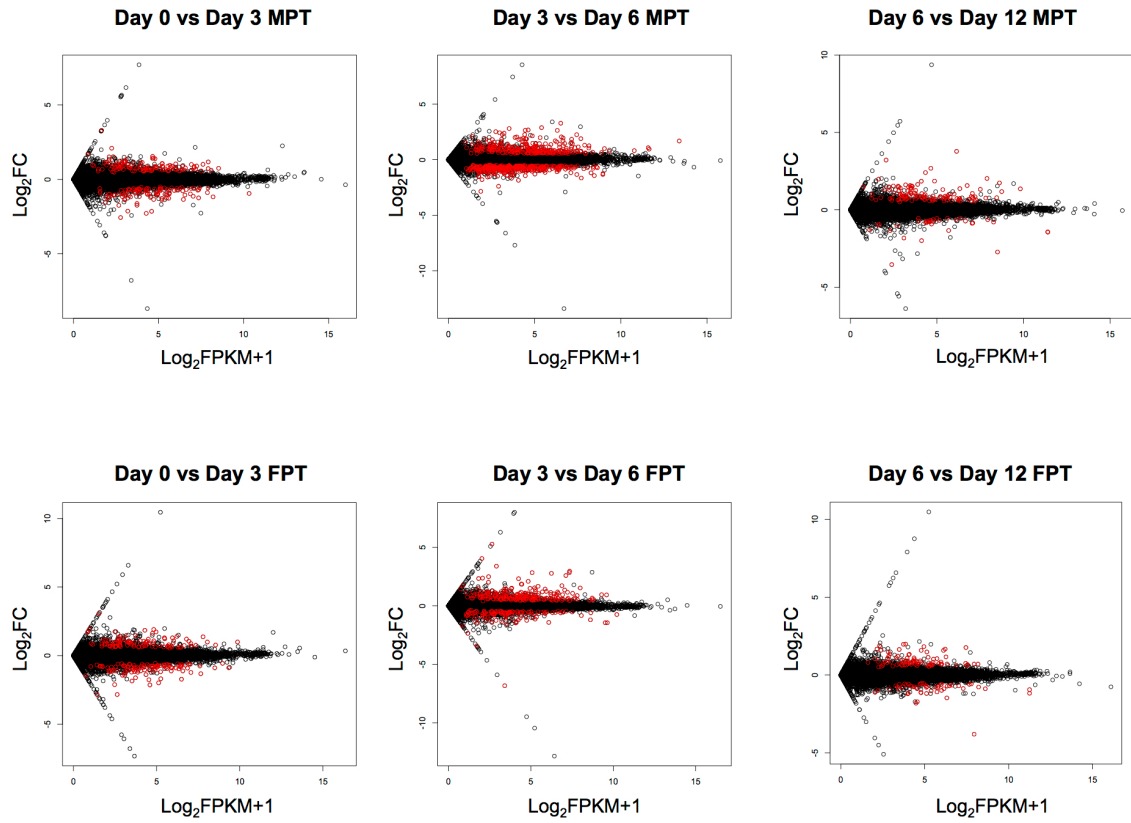**b**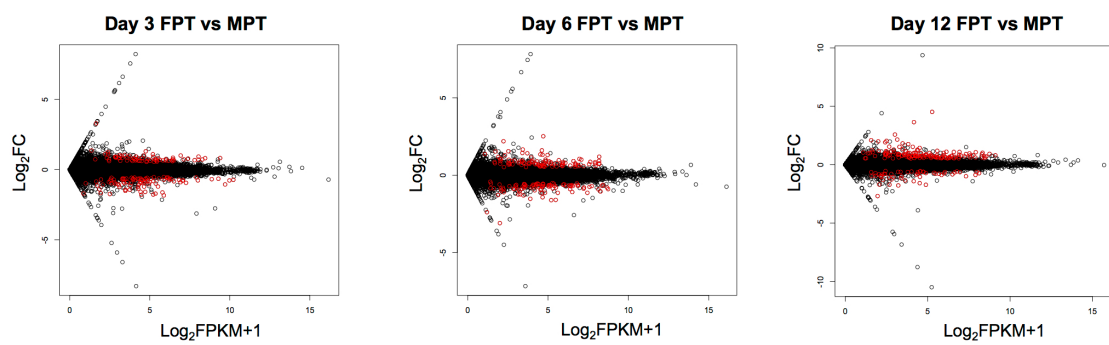

Supplement: Additional file 2: — Development-dependent DEGs and Sex-dependent DEGs in alligator transcriptomic profiles. (a) MA plot, with gene expression values expressed as log2FPKM and fold change expressed as Log2FC, is constructed using differential expression analysis between each time point (Day 0-3, Day 3–6, Day 6-12). (b) MA plot, with gene expression values expressed as log2FPKM and fold change expressed as Log2FC, is constructed using differential expression analysis results between FPT and MPT (30 °C vs 33.5 °C) for Day 3 FPT vs Day 3 MPT, Day 6 FPT vs Day 6 MPT, and Day 12 FPT vs Day 12 MPT. For both MA plot, 20,181 genes, based on NCBI genome annotation, were examined for differential expression. Red dots indicate significantly up- or down-regulated genes at FDR < 0.01. (PDF 1359 kb) [file 12864_2016_2396_MOESM2_ESM.pdf]

## Slide 1
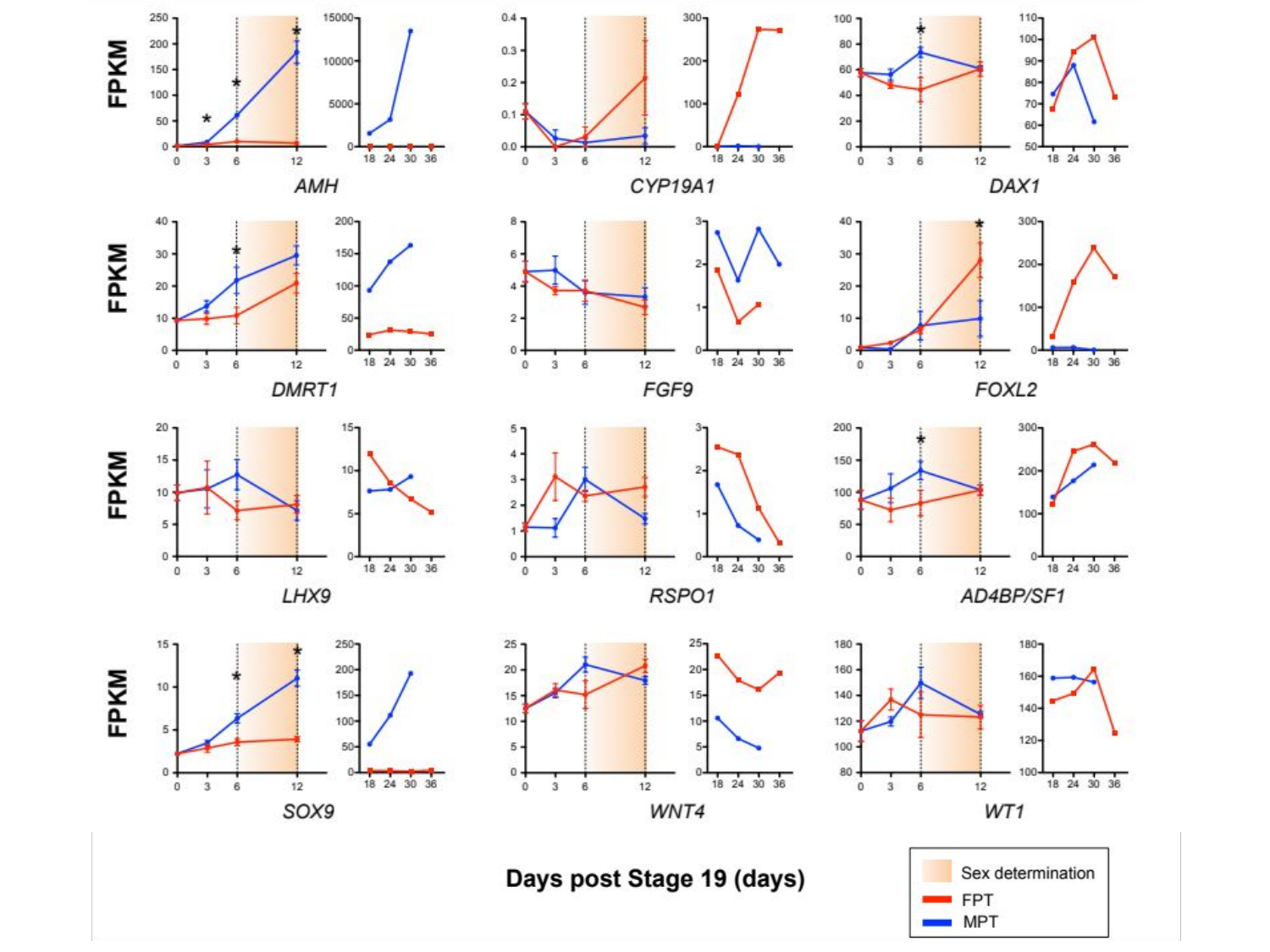

Supplement: Additional file 5: — Expression profiles of major genes involved in sex differentiation. Expression profiles of various major genes involved in gonadal sex determination in model organisms are displayed based on FPKM values from RNA-seq analysis under FPT conditions (indicated in red) and MPT conditions (indicated in blue). Predicted onset of sexual differentiation is indicated in orange background; * FDR ≤0.01. (PPT 968 kb) [file 12864_2016_2396_MOESM5_ESM.ppt]

## Slide 1
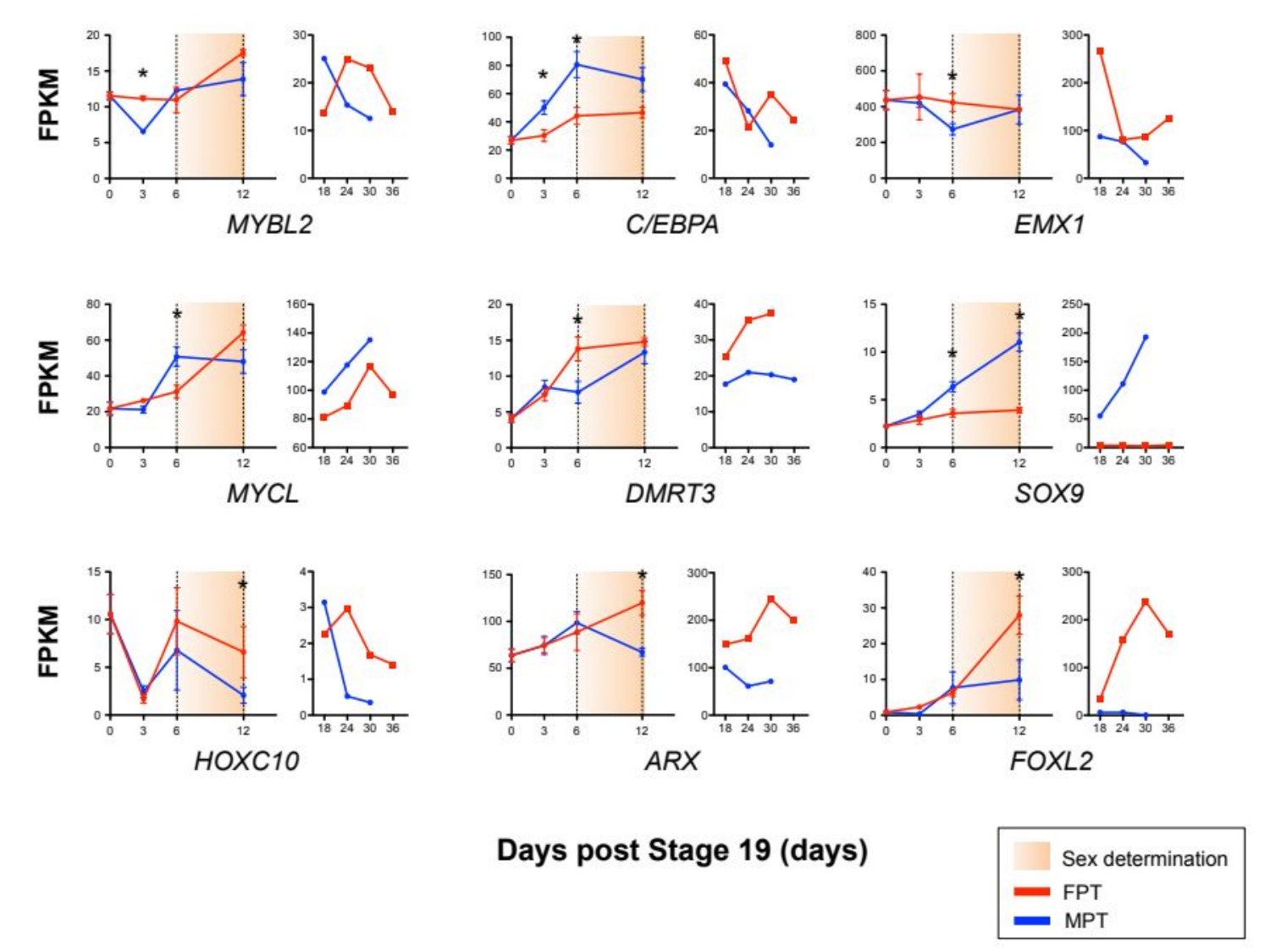

Supplement: Additional file 7: — Expression profiles of candidate transcription factors involved in sex differentiation. Expression profiles of candidate transcription factors involved in gonadal sex determination are displayed based on FPKM values from RNA-seq analysis under FPT conditions (indicated in red) and MPT conditions (indicated in blue). Predicted onset of sexual differentiation is indicated in orange background; * FDR ≤0.01. (PPT 344 kb) [file 12864_2016_2396_MOESM7_ESM.ppt]
